# Editorial: Metal nanoparticles in cancer: detection, diagnosis, therapy and their pharmacological assessment

**DOI:** 10.3389/fonc.2023.1344197

**Published:** 2024-01-04

**Authors:** Massimiliano Peana, Alexandros G. Georgakilas

**Affiliations:** ^1^ Department of Chemical, Physical, Mathematical and Natural Sciences, University of Sassari, Sassari, Italy; ^2^ DNA Damage Laboratory, Physics Department, School of Applied Mathematical and Physical Sciences, National Technical University of Athens, Athens, Greece

**Keywords:** cancer theragnostic, metal nanoparticle (MNP), cancer, toxicological assessment, cancer treatment, pharmacological assessment

Metal nanoparticles (MNPs) represent a promising class of nanomaterials for various biomedical applications, especially in the field of chemotherapy (1). MNPs can be designed to carry and deliver therapeutic agents, such as chemotherapy drugs, to specific targeted sites within the body. This targeted drug delivery minimizes damage to healthy tissues and enhances the therapeutic efficacy of the treatment (precision medicine) (2).

The MNPs surface can be easily modified to improve the biocompatibility and to attach specific targeting molecules. This allows for the selective targeting of cancer cells, increasing once more the precision of the treatment. MNPs can be employed in conjunction with conventional chemotherapy drugs to induce synergistic effects. This approach has the potential to enhance the overall therapeutic outcome while reducing the dosage of conventional drugs and associated side effects (2).

Some MNPs, such as gold nanoparticles, have inherent therapeutic properties (3). They can interact with light to generate heat (photothermal therapy) or be used in photodynamic therapy, where they produce reactive oxygen species (ROS) upon exposure to light, leading to cell death. Moreover, some MNPs exhibit unique optical and magnetic properties that can be exploited for imaging and diagnostic purposes. This is particularly valuable for cancer diagnosis and monitoring treatment progress. Certain MNPs, like gold and silver nanoparticles, are known for their biocompatibility. This is crucial for their use in medical applications, ensuring minimal toxicity and adverse effects (4).

In summary, owing to their distinctive characteristics, including high versatility, MNPs can be utilized across the entire spectrum of cancer management, encompassing early detection, diagnosis, and treatment. Theranostic, stimulus-responsive MNPs can not only be designed for tumor targeting, drug delivery, and enhancement of conventional cancer immunotherapy, but also to create biosensors, increase imaging performance, and repair damaged tissue.

Despite these advantages and the enormous potential of MNPs, their high reactivity due to their small size and consequent large surface area could induce unwanted side effects related to their interaction with non-specific random cellular targets, thus posing challenges such as long-term toxicity and potential accumulation in organs (5). Although the importance of MNPs as promising theranostic agents is indisputable, their translation into clinical practice is hampered by a number of aspects related to unclear toxicity in humans and the lack of in-depth *in vivo* studies in reliable animal models (6). These issues must be addressed in order to successfully translate MNPs into clinical applications.

Therefore, this Research Topic “*Metal Nanoparticles in Cancer: Detection, Diagnosis, Therapy and Their Pharmacological Assessment*” aimed to bring to light the potential application of MNPs in cancer treatment, besides their diagnostic and therapeutic (theranostic) properties, whilst considering the factors involved in their toxicological assessment, such as biodistribution, clearance and pharmacokinetics, metabolism mechanisms, long-lasting toxicity, and mechanism of pharmacological activities. A total 4 articles by 37 authors in the fields of chemistry, cancer biology, and pharmacology, have been collected.

In this Research Topic, the research article by Dun et al. evaluated the *in vivo* application and photothermal ablation effects and mechanism of copper sulfide nanoparticles (CuS NPs) in hepatocellular carcinoma (HCC), the sixth most common cancer and the third leading cause of cancer-related deaths globally in the global cancer burden in 2020 (7). As a photothermal agent, sheet-like CuS-BSA NPs with a particle size of 30 nm, synthesized using bovine serum albumin (BSA) as a biological modifier, have been shown to effectively inhibit tumor growth in H22 tumor-bearing mice under 980 nm NIR. CuS-BSA NPs-mediated photothermal therapy caused coagulative necrosis and up-regulated the expression of apoptosis proteins including cleaved caspase-3 and cleaved caspase-9, which inhibited tumor growth by inducing apoptosis. In the evaluation of *in vivo* toxicity, when the concentration of CuS-BSA NPs was in the range of 1,800-7,200 µg/Kg, the thrived and showed positive growth, and there were no observed pathological alterations in the liver, spleen, or kidney. CuS-BSA NPs emerge as a promising candidate in the realm of photothermal therapy for cancer.

The research paper of Yang et al. described the synthesis and characterization of a novel nanoplatform that combines chemo-photothermal-targeted anti-cancer therapy. The innovative drug delivery system has been prepared by combining chemotherapy and photothermal therapy for liver cancer, using one of the metal-organic frameworks (MOFs), Zn-Co ZIF. Taking advantage of its pH-sensitive property, the material can be effectively cleaved under acidic conditions in the tumor microenvironment, releasing Co^2+^ and the adriamycin hydrochloride drug DOX. Co^2+^ acts as a catalyst for H_2_O_2_, generating O_2_ to alleviate the tumor hypoxic environment, while the released DOX serves for chemotherapy. Furthermore, to enhance biocompatibility, chitosan was applied as an outer coating to the NPs. Additionally, the incorporation of gold NPs imparts excellent photothermal sensitivity for photothermal therapy and the attachment of SH-RGD on the NPs surface ensures effective tumor targeting, minimizing harm to healthy tissues. *In vitro* assessments, including cytotoxicity and confocal experiments, validate the ZD-CAR nano-drug delivery platform’s efficacy, offering a novel approach for combined chemotherapy and photothermal treatment with promising applications in anti-cancer therapy.

The demand for the advancement of photodynamic therapy (PDT) efficiency has led to a quest for efficient photosensitizers with attributes such as high singlet oxygen quantum yield, robust fluorescent emission, excellent photostability, and specific organelle targeting. In the study by Xu et al., a novel two-photon photosensitizer, chlorophenyl thiophene axially substituted silicon (IV) phthalocyanine (CBT-SiPc), was designed and synthesized. CBT-SiPc exhibited specific targeting of lysosomes in living cells and demonstrated good biocompatibility. Moreover, the photosensitizer displayed high efficiency in generating singlet oxygen and achieved remarkable PDT efficiency in MCF-7 breast cancers under irradiation. The innovative CBT-SiPc holds significant promise for applications in lysosome-targeted and two-photon bioimaging-guided photodynamic cancer therapy. To improve radioPDT efficiency, Azad et al. devoleped a novel variant of pegylated poly-lactic-co-glycolic (PEG-PLGA) encapsulated nanoscintillators (NSCs), coupled with a highly efficient ruthenium-based photosensitizer (Ru/radioPDT). The NP showed size of 120 nm, a polydispersity index (PDI) of less than 0.25, and a high NSCs loading efficiency exceeding 90%. *In vitro* assessments demonstrated the NP’s accumulation within the cytosolic structures of the endoplasmic reticulum and lysosome. The therapeutic efficacy of Ru/radioPDT was evaluated using PC3 cell viability and clonogenic assays, revealing minimal cell toxicity until activated by radiation, resulting in significant cancer cell kill compared to radiation alone. In comparison to protoporphyrin IX-mediated radioPDT (PPIX/radioPDT), Ru/radioPDT exhibited a higher capacity for singlet oxygen generation while maintaining a comparable cytotoxic effect on PC3 cells.

In summary, these investigations emphasize different aspects of the evolving strategies in utilizing MNPs, illustrating their potential importance in the context of cancer therapies and the significance of toxicological assessment for the future clinical application of the most promising candidates.

## Author contributions

MP: Conceptualization, Funding acquisition, Supervision, Writing – original draft, Writing – review & editing. AG: Conceptualization, Funding acquisition, Writing – review & editing.
